# Eicosapentaenoic acid administration ameliorates the progression of liver fibrosis after laparoscopic Kasai portoenterostomy

**DOI:** 10.1007/s00383-024-05800-2

**Published:** 2024-08-21

**Authors:** Wataru Sumida, Takahisa Tainaka, Chiyoe Shirota, Satoshi Makita, Hizuru Amano, Akihiro Yasui, Takuya Maeda, Daiki Kato, Yosuke Goda, Hiroki Ishii, Kazuki Ota, Guo Yaohui, Liu Jiahui, Akinari Hinoki, Hiroo Uchida

**Affiliations:** Department of Pediatric Surgery, Nagoya University Graduate School of Medicine, 65 Tsurumai-cho, Showa-ku, Nagoya, 466-8550 Japan

**Keywords:** Biliary atresia, Eicosapentaenoic acid, Liver fibrosis, Inflammation, Polyunsaturated fatty acid

## Abstract

**Purpose:**

Biliary atresia (BA) poses a persistent challenge characterized by ongoing liver inflammation and subsequent fibrosis even after the clearance of jaundice (COJ). This study aimed to evaluate the therapeutic potential of eicosapentaenoic acid (EPA) in alleviating liver inflammation and limiting fibrosis during the post-COJ phase of BA.

**Methods:**

Among the BA patients undergoing laparoscopic Kasai portoenterostomy (lapKP) between December 2016 and October 2021, EPA (20–40 mg/kg/day) was administered orally to those whose parents consented. The study included patients from January 2014 to October 2021, classifying them into two groups: EPA-treated (Group E) and untreated (Group N). Their liver fibrosis and clinical course at 1 and 2 years post-lapKP were compared.

**Results:**

Group E consisted of 25 patients, while Group N comprised 32 patients. Twenty-one patients in Group E and 25 patients in Group N achieved COJ (*p* = 0.74). Among jaundice-free patients at 1 and 2 years post-lapKP, Group E exhibited significantly lower M2BPGi levels and platelet counts, and Group E showed a significant reduction in Aminotransferase-to-Platelet Ratio Index (APRI) at 2 years post-lapKP.

**Conclusion:**

Although EPA administration did not improve COJ, it attenuated the progression of liver fibrosis during the 2 years following lapKP in jaundice-free patients.

(200/200Words).

## Introduction

Biliary atresia (BA) is a condition marked by progressive fibrosis and obstruction of the biliary tree due to inflammation of unknown origin. Untreated BA leads to cholestasis, resulting in gradual cirrhosis and liver failure, with survival beyond 2 years being uncommon [1].

Kasai portoenterostomy (KP) is a surgical procedure aimed at establishing bile drainage. It involves dissection of the porta hepatis tissue to reveal residual microscopic bile ductile, followed by anastomosis of the jejunum in a Roux-Y fashion to the porta hepatis to collect bile secretion [2]. Achieving clearance of jaundice (COJ) through KP is the primary means of survival for BA patients with their own liver. Failure to attain COJ post-KP leads to ongoing liver cirrhosis, necessitating liver transplantation (LT) for survival.

Persistent inflammation persists around Glisson’s capsule of the liver even after achieving COJ following KP BA patients. Recent studies have highlighted significantly elevated levels of inflammatory cytokines, such as IL-8, in BA patients [3]. Consequently, liver fibrosis continues to advance even after COJ is achieved through KP, with some BA patients eventually requiring LT post-COJ [4].

Emerging research indicates that metabolic byproducts of polyunsaturated fatty acids (PUFA) regulate the immune system’s communication network. PUFA comprises essential fatty acids categorized into two families: n-3 (or omega-3) and n-6 (or omega-6). The n-3 PUFA family serves as a precursor to anti-inflammatory cytokines, while the n-6 PUFA family is associated with pro-inflammatory cytokines [5].

In a previous study, we observed that BA patients who achieved COJ had a lower n-3/n-6 ratio compared to healthy individuals or BA patients post-LT [6]. A low n-3/n-6 ratio may predispose patients to sustained inflammation. Therefore, we hypothesized that administering n-3 PUFA could alleviate inflammation and conducted a prospective study administering EPA, a type of n-3 PUFA, to BA patients [7].

This study presents longer term results from a larger cohort in a prospective trial.

## Methods

A prospective clinical study was conducted to administer EPA to postoperative patients with BA between December 2016 and October 2021, with approval from the Ethics Committee (Ref No. 2016–0306). Upon obtaining informed written consent, EPA was orally administered to BA patients starting from the seventh day after surgery. EPA administration continued until LT was planned or the patient’s parent requested discontinuation of the study. The dosage of EPA was set at 30 mg/kg/day, with a permissible range of ± 10 mg/kg/day (i.e., 20–40 mg/kg/day). The specific medication used was Epadel S (Mochida Pharmaceutical Co., Ltd., Tokyo, Japan). The EPA dose for present trial was considered from the usual dosage of Epadel S for adults, which was 1800 mg–2700 mg/day. The decision to administer EPA was made by the patient’s parents after receiving a detailed explanation about the trial and was not based on random assignment.

To assess the effects of EPA administration, we reviewed BA patients who underwent laparoscopic Kasai portoenterostomy (lapKP) at our institution from January 2014 to October 2021, with approval from the Ethics Committee (Ref No. 2022–0341). The eligible patients were divided into two groups: the EPA administration group (Group E) and the non-administration group (Group N), i.e., the patients with BA operated from December 2016 to October 2021 were divided into Group E and Group N according to EPA administration. Patients from January 2014 to November 2016 were all assigned Group N. Patients in whom EPA administration was discontinued due to parental request or inability to take oral medication were excluded from the study. No patients refused the review itself.

Comparison was made between the two groups regarding the attainment of COJ. Subsequently, among patients with jaundice-free native liver survival at 1 or 2 years post-initial lapKP, various aspects including clinical progression, laboratory findings, and management of esophageal varices (EV) were compared between the groups. Laboratory data were sourced from records obtained 1 or 2 years following the initial lapKP. However, if laboratory tests could not be conducted within 3 months of the scheduled date, the data were considered missing. The degree of liver fibrosis was assessed using the Mac-2 binding protein sugar chain modified isomer (M2BPGi) and the aspartate Aminotransferase-to-Platelet Ratio Index (APRI). APRI was calculated as follows: aspartate transaminase (AST) value (IU/L)/upper normal limit of AST in our hospital, i.e., 30 IU/L)/platelet count (10^9^/L). Serum fatty acids were analyzed by LSI Medience Corporation (Tokyo, Japan), and the n-3/n-6 ratio was determined by sum of concentration of all n-3 PUFAs/sum of concentration of all n-6 PUFAs.

During the review period, no alterations were made in postoperative treatment apart from EPA administration, which included a 2-day course of antibiotics for postoperative prophylaxis; initiation of oral intake on the third postoperative day; intravenous administration of 200 mg/day of dehydrocholate for 4 days post-surgery as a cholagogue, followed by oral administration of 20 mg/kg/day of ursodeoxycholate; initiation of prednisolone on the fifth postoperative day at an initial dosage of 4 mg/kg/day, with subsequent reduction to 2, 1, and 0.5 mg/kg/day every 5 days until termination of administration. In case of high-grade fever, antibiotic administration commenced considering cholangitis. If jaundice levels rose, pulse therapy with steroids or reoperation was contemplated.

Observation and treatment of EV were carried out by gastroenterologists at regular intervals and in the event of gastrointestinal bleeding. Appropriate intervention was undertaken if the red color sign was positive, at the discretion of the gastroenterologist.

Statistical analysis utilized Fisher’s exact test, Mann–Whitney U test, and Friedman test with Bonferroni correction, with *p* < 0.05 deemed statistically significant.

The authors declare no conflicts of interest.

## Results

During the study period, lapKP was performed on 60 patients at our hospital. After excluding 3 patients who chose to discontinue treatment as per parental decision, a total of 57 patients were included in the study cohort. Upon group assignment, 25 patients were categorized into Group E, while 32 patients were placed in Group N. There were no statistically significant differences observed in the demographic characteristics of patients between the two groups (Table 1).

**Table 1:** Summary of demographic characteristics of the enrolled patients

	EPA group		nonEPA group		*p* value
Number of cases	25		32		
Male/female	6/19		10/22		0.77
Age at the first lapKP (day)	56	(36–69)	56	(46–74)	0.36
Opetative time (min)	369	(308–408)	325	(288–386)	0.18
BW at the first lapKP (kg)	4.25	(3.83–4.85)	4.34	(4.07 –4.80)	0.38
Bleeding (mL/kg)	5.23	(3.84–12.44)	5.87	(1.84–9.22)	0.48
Number of cases received intraoperative blood transfusion	3/25		3/32		1

In terms of COJ, 21 patients in Group E and 25 patients in Group N achieved COJ at least once, with no significant difference in the COJ attainment ration between the two groups (*p* = 0.74). In Group E, 16 (76.2%) and 14 (66.7%) patients maintained jaundice-free native liver survival at 1 and 2 years post-lapKP, respectively. Conversely, in Group N, 20 (80%) and 18 (72%) patients sustained jaundice-free native liver survival at 1 and 2 years post-lapKP, respectively. The rates of jaundice-free native liver survival at 1 and 2 years post-lapKP did not exhibit significant differences between the two groups (*p* = 1.0 in both comparisons).

There were instances of unavailable data at appropriate time points, including one patient each at 1 and 2 years post-lapKP in Group E and two patients at 1 year post-lapKP in Group N. These cases were excluded from subsequent numerical analyses. Therefore, data were available for 15 and 13 patients at 1 and 2 years post-lapKP in Group E, respectively, while 18 and 18 patients had available data at 1 and 2 years post-lapKP in Group N.

**Fig. 1:**
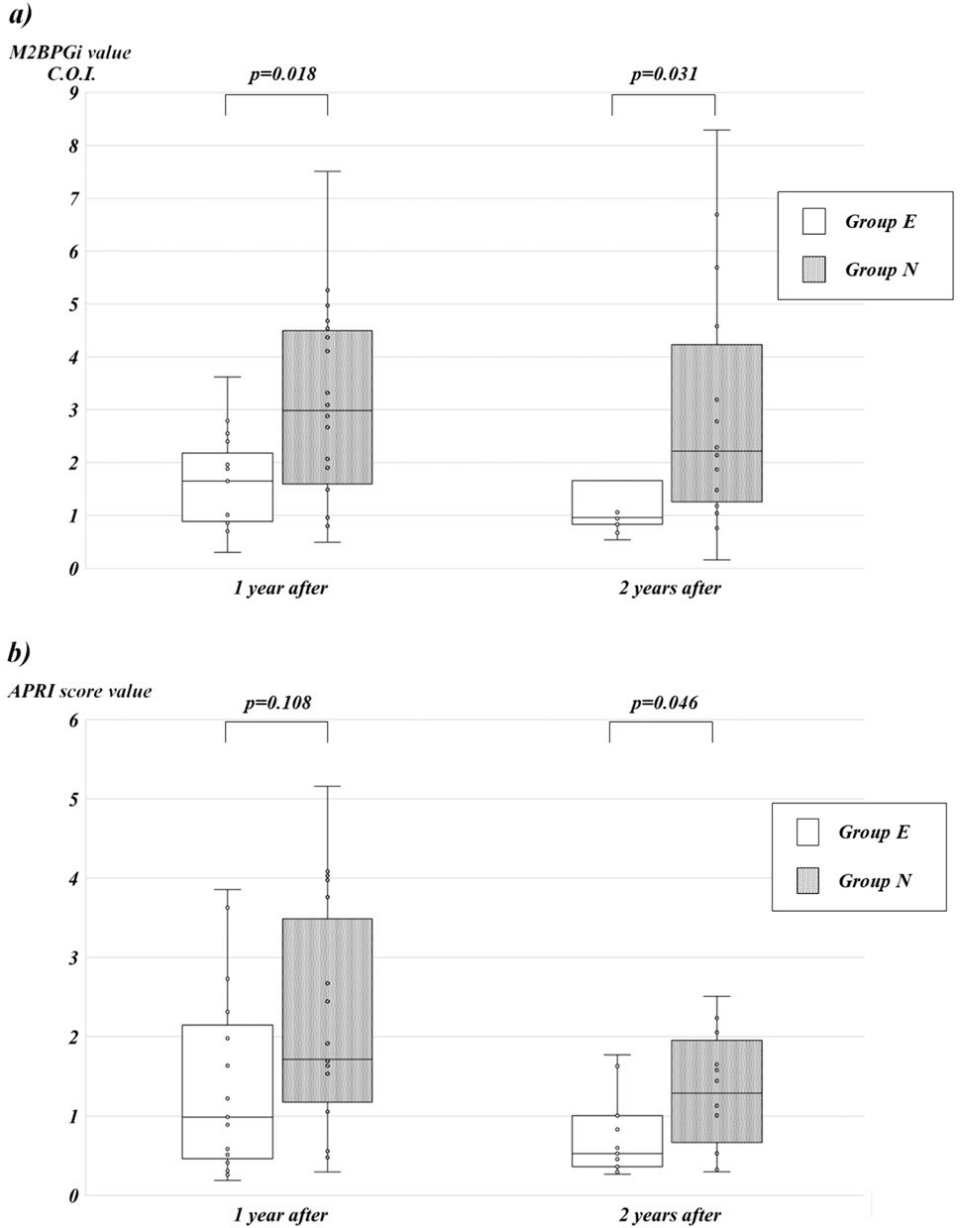
Box-and-Whisker plots illustrating M2BPGi (**a**) and APRI (**b**) comparisons between Group E and Group N of the study participants. White boxes represent Group E, while gray boxes represent Group N. The respective values for each group within the whisker line are indicated by dots. There was one outlier for both M2BPGi and APRI in the results of 2 years after in the Group N, but they were not plotted in this figure

The comparison of laboratory data, detailed in Table 2, indicated that the platelet count in Group E exceeded that of Group N after both 1 and 2 years post-lapKP. Similarly, the choline esterase (ChE) level in Group E showed an increase compared to Group N solely after 1 year post-lapKP. The median value of M2BPGi was 1.65 (range 0.30–3.62) in Group E and 3.00 (range 0.49–7.51) in Group N after 1 year post-lapKP, and 0.96 (range 0.54–4.13) in Group E and 2.22 (range 0.16–10.25) in Group N after 2 years. The M2BPGi value in Group E was significantly lower in both comparisons (*p* = 0.018, 0.031, respectively) (Fig. 1a). The median APRI value was 0.99 (range 0.19–3.86) in Group E and 1.71 (range 0.30–5.16) in Group N after 1 year post-lapKP, and 0.53 (range 0.27–3.53) in Group E and 1.29 (range 0.30–22.60) in Group N after 2 years. The APRI value in Group E was significantly lower solely after 2 years post-lapKP (p = 0.108, 0.046, respectively) (Fig. 1b).

**Table 2:** Comparison of laboratory data among enrolled patients after 1 year and 2 years post-lapKP

		Group E		Group N		*p* value
Post op 1 year						
AST	IU/L	75	(50–126)	89	(58–128)	0.60
ALT	IU/L	66	(28–100)	53	(44–94)	0.77
T-B	mg/dL	0.6	(0.5–0.8)	0.7	(0.6–1.0)	0.12
D-B	mg/dL	0.1	(0.1–0.1)	0.1	(0.1–0.3)	0.09
GGT	IU/L	121	(56–349)	145	(67–178)	0.91
ChE	IU/L	310	(281–362)	242	(201–280)	0.01
TBA	mg/dL	65.8	(25.0–148.0)	100.9	(52.0–234.1)	0.26
Plt	× 10^9/L	287	(199–459)	166	(148–243)	0.005
Post op 2 years						
AST	IU/L	51	(41–78)	59	(40–84)	0.70
ALT	IU/L	30	(22–65)	48	(28–58)	0.66
T-B	mg/dL	0.7	(0.4–0.8)	0.7	(0.5–0.9)	0.29
D-B	mg/dL	0.1	(0.0–0.1)	0.1	(0.1–0.1)	0.19
GGT	IU/L	73	(43–152)	53	(28–85)	0.40
ChE	IU/L	345	(280–372)	264	(230–346)	0.07
TBA	mg/dL	39.4	(18.6–64.0)	64.9	(34.0–118.9)	0.33
Plt	× 10^9/L	314	(259–323)	170.5	(134 201)	0.003

**Fig. 2:**
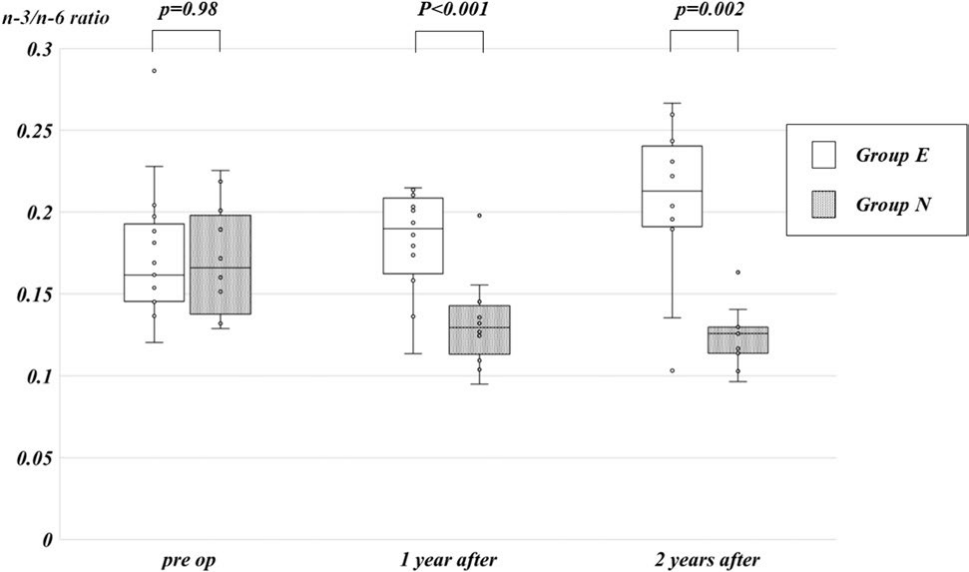
Box-and-Whisker plot displaying the total n-3/n-6 ratio and its comparison between Group E and Group N of the enrolled patients. White boxes denote Group E, whereas gray boxes denote Group N. The respective values for each group containing outlier are indicated by dots

The median total n-3/n-6 ratios of Group E before lapKP and 1 and 2 years after lapKP were 0.16 (range 0.12–0.29), 0.19 (range 0.11–0.21), and 0.21 (0.10–0.27), respectively. Meanwhile, those of Group N at the same time points were 0.17 (range 0.13–0.23), 0.13 (range 0.09–0.20), and 0.13 (range 0.10–0.16), respectively. There was no significant difference in the preoperative total n-3/n-6 ratio between Groups E and N (*p* = 0.98). However, the total n-3/n-6 ratio in Group E was significantly higher than that in Group N at 1 and 2 years post-lapKP (*p* < 0.001, *p* = 0.002, respectively) (Fig. 2).

In Group N, two cases required treatment for EV within 1 year and five cases within 2 years, whereas no cases in Group E required such intervention within the same timeframe. The frequency of EV treatment requirements showed no significant differences between the two groups at both 1 and 2 years post-lapKP (*p* = 0.49, *p* = 0.058, respectively). There were no incidents of gastrointestinal bleeding within 2 years among the enrolled patients.

## Discussion

BA is a liver disorder occurring in newborns and early infancy, characterized by its rarity, with reported incidences ranging from 1 in 5000–20,000 live births, varying significantly among countries with population-based data [8]. Although its precise cause remains elusive, KP stands as the sole treatment option for BA patients to sustain with their native liver, representing the standard initial treatment for BA in Japan presently [9].

The absence of COJ leads to progressive cirrhosis and liver failure, resulting in a limited number of patients surviving beyond 2 years without requiring LT [10]. Therefore, pediatric surgeons frequently encounter challenges in achieving COJ in BA patients. However, COJ does not always ensure long-term native liver survival for BA patients. Indeed, a fraction of patients still necessitate LT even after jaundice resolution. Reports from East Asia, including Japan, China, and Korea, note a discrepancy of 10–20% between the rates of jaundice clearance and long-term native liver survival [4], often attributed to cases requiring LT post-COJ.

Criteria for LT in post-KP BA patients encompass liver cirrhosis, liver failure, gastrointestinal bleeding, growth retardation, pruritus, hepatopulmonary syndrome, and recurrent cholangitis [11]. Nonetheless, accurate proportions for each indication remain scant in the literature. One study indicated that 83% of LT cases were linked to jaundice, whereas in non-jaundice cases, 55% were associated with hepatopulmonary syndrome, 27% with cholangitis, and 9% with gastrointestinal bleeding [12]. Another report detailed indications for LT in cases without jaundice, with 44% attributed to cholangitis, 30% to portal hypertension, and 23% to gastrointestinal bleeding [13]. These findings indicate that symptoms associated with portal hypertension contribute substantially to LT indications in BA patients following COJ achievement. Thus, mitigating portal hypertension in BA patients could potentially enhance native liver survival in this population.

This observation underscores that liver fibrosis progresses in patients with after the disappearance of jaundice, leading to continued advancement of portal hypertension. This phenomenon seems to be driven by inflammation around Glisson’s capsule, a distinctive feature of BA. Recent studies have highlighted significantly elevated levels of inflammatory cytokines, such as IL-8, in BA patients [3]. Persistent inflammation around Glisson’s capsule, despite achieving COJ, can trigger liver fibrosis and ultimately culminate in cirrhosis due to elevated cytokine levels.

In a prior study, we demonstrated that even in BA patients who achieved COJ, the ratio of n-3/n-6 PUFA decreased. Specifically, the median n-3/n-6 ratio was 0.11 in BA patients who survived with their native liver without jaundice, while the median n-3/n-6 ratio for normal controls was 0.16 [6]. In our current investigation, the median n-3/n-6 ratios at 1 and 2 years post-surgery in Group N, which did not receive EPA, were 0.13 and 0.13, respectively. Conversely, the median n-3/n-6 ratios at 1 and 2 years in Group E, treated with EPA, were 0.19 and 0.21, respectively. The n-3/n-6 ratio values in Group N were lower than those of normal controls reported previously and resembled those of jaundice-free BA patients with native liver, although statistical comparisons are unavailable. Conversely, the n-3/n-6 ratio of Group E, receiving EPA treatment in the current study, was higher than that of the previously reported normal controls.

It has been established that n-3 PUFA are metabolized to eicosanoids capable of suppressing inflammation, while n-6 PUFA are metabolized into eicosanoids that can promote inflammation [5]. Additionally, studies have indicated that n-3 PUFA suppresses the expression of IL-8 induced by inflammatory stimuli [14]. This indicates that inflammation may persist in patients even after achieving COJ.

Given these findings, we hypothesized that supplementation with EPA, a type of n-3 PUFA, could alleviate liver fibrosis in patients with BA by reducing the inflammatory response. Previously, we reported short-term results of EPA supplementation in BA patients, where oral administration of EPA suppressed liver fibrosis in patients without jaundice 6 months after lapKP [7]. The current study presents the results of EPA supplementation in a larger cohort over an extended duration, specifically at 1 and 2 years post-lapKP. The findings were consistent with our previous report, indicating statistically significant reductions in M2BPGi and APRI, both indicators of liver fibrosis and cirrhosis.

M2BPGi is a juxtacrine-acting messenger between hepatic stellate cells and Kupffer cells during liver fibrosis, reflecting the activation of hepatic stellate cells and playing a pivotal role in fibrosis progression [15]. APRI was initially proposed to evaluate hepatic fibrosis in patients with chronic hepatitis C using standard laboratory tests [16]. Both M2BPGi and APRI are effective in evaluating the degree of liver fibrosis in BA patients [17, 18].

APRI is a metric that reflects thrombocytopenia based on its formula. Historically, thrombocytopenia was attributed to increased platelet pooling in the enlarged spleen due to portal hypertension. However, recent studies proposed that reduced circulating thrombopoietin levels and diminished response to thrombopoietin are the primary causes of thrombocytopenia in cirrhosis patients. This results from enhanced degradation of thrombopoietin by platelets pooled in the spleen [19]. Therefore, APRI has been suggested as a better indicator of portal hypertension rather than liver fibrosis [20]. According to our findings, significant differences were observed in M2BPGi after 1 and 2 years, whereas significant differences were noted in APRI only after 2 years. This discrepancy may be attributed to the temporal progression, where portal hypertension advances subsequent to liver fibrosis.

In our earlier study, it was noted that patients with EV who experienced bleeding or necessitated treatment exhibited notably lower platelet counts and ChE values compared to those without EV during the follow-up of BA patients [21]. These findings imply that reduced platelet counts and ChE values signify more severe portal hypertension. Our present study reveals that platelet count and ChE values were significantly lower in Group N compared to Group E. Additionally, only patients in Group N required EV treatment, although the frequency did not reach statistical significance. These outcomes underscore that portal hypertension was more advanced in Group N than in Group E.

In our previous study, it was found that the n-3/n-6 ratios were notably higher in BA patients who received eicosapentaenoic acid (EPA) supplementation compared to those without EPA administration at the 6-month mark after lapKP [7]. This current report indicates a sustained trend toward elevated n-3/n-6 ratios in Group E compared to Group N at 1 and 2 years post-lapKP. This suggests that EPA supplementation may mitigate the progression of liver fibrosis by maintaining a higher n-3/n-6 ratio.

However, there was no significant difference observed between Groups E and N regarding the likelihood of achieving COJ. The attainment of COJ in BA patients entails consideration of various factors, including the patient's original anatomy and surgical precision. Thus, the administration of EPA alone may not exert a significant influence on COJ but could potentially ameliorate the advancement of liver fibrosis following COJ achievement.

A limitation of the present study is that it is not a randomized study. As a result, it is challenging to completely exclude biases, such as the assignment of a large number of BA cases with poor prognoses to Group N. Due to the biases, it is difficult to prove with certainty that the results of this study were caused by the effects of EPA. However, it is unlikely that there was significant bias in the allocation of patients. The reason for this is that the exclusion criteria (achievement of COJ, recurrence of jaundice, etc.) were identical in both groups. Furthermore, the ratio of cases excluded between the two groups was comparable. Additionally, the postoperative management of lapKP was consistent throughout the study period, and there was no difference based on the timing or assigned group. Therefore, it is unlikely that these limitations significantly influenced the results.

## Conclusion

The administration of EPA to patients with BA did not enhance the likelihood of achieving COJ. However, when administered solely to jaundice-free patients, EPA significantly mitigated the advancement of liver fibrosis and diminished portal hypertension in BA patients. Additionally, EPA supplementation can attenuate inflammation around Glisson’s capsule, which persists even after COJ in BA patients, thereby reducing liver fibrosis.

## Data Availability

No datasets were generated or analysed during the current study.

## References

[1] Sumida W, Uchida H, Tanaka Y, Tainaka T, Shirota C, Murase N, Oshima K, Shirotsuki R, Chiba K (2017) Review of redo-Kasai portoenterostomy for biliary atresia in the transition to the liver transplantation era. Nagoya J Med Sci 79:415–420. 10.18999/nagjms.79.3.415 28878446 10.18999/nagjms.79.3.415 PMC5577027

[2] Ohi R (2001) Surgery for biliary atresia. Liver 21:175–182. 10.1034/j.1600-0676.2001.021003175.x 11422780 10.1034/j.1600-0676.2001.021003175.x

[3] Dong R, Zheng S (2015) Interleukin-8: a critical chemokine in biliary atresia. J Gastroenterol Hepatol 30:970–976. 10.1111/jgh.12900 25611432 10.1111/jgh.12900

[4] Chung PHY, Zheng S, Tam PKH (2020) Biliary atresia: east versus west. Semin Pediatr Surg 29:150950. 10.1016/j.sempedsurg.2020.150950 32861448 10.1016/j.sempedsurg.2020.150950

[5] Das UN (2006) Essential fatty acids: biochemistry, physiology and pathology. Biotechnol J 1:420–439. 10.1002/biot.200600012 16892270 10.1002/biot.200600012

[6] Sumida W, Kaneko K, Ono Y, Tainaka T, Ando H (2009) Different polyunsaturated fatty acid profiles in patients with biliary atresia after successful Kasai operation and liver transplantation. Pediatr Surg Int 25:255–259. 10.1007/s00383-009-2324-z 19184057 10.1007/s00383-009-2324-z

[7] Sumida W, Uchida H, Tainaka T, Shirota C, Hinoki A, Kato T, Yokota K, Oshima K, Shirotuki R, Chiba K, Tanaka Y (2018) Oral administration of eicosapentaenoic acid suppresses liver fibrosis in postoperative patients with biliary atresia. Pediatr Surg Int 34:1059–1063. 10.1007/s00383-018-4313-6 30056480 10.1007/s00383-018-4313-6

[8] Schreiber RA, Harpavat S, Hulscher JBF, Wildhaber BE (2022) Biliary atresia in 2021: epidemiology, screening and public policy. J Clin Med. 10.3390/jcm11040999 35207269 10.3390/jcm11040999 PMC8876662

[9] Nio M (2017) Japanese biliary atresia registry. Pediatr Surg Int 33:1319–1325. 10.1007/s00383-017-4160-x 29039049 10.1007/s00383-017-4160-x

[10] Bates MD, Bucuvalas JC, Alonso MH, Ryckman FC (1998) Biliary atresia: pathogenesis and treatment. Semin Liver Dis 18:281–293. 10.1055/s-2007-1007164 9773428 10.1055/s-2007-1007164

[11] Kasahara M, Umeshita K, Sakamoto S, Fukuda A, Furukawa H, Uemoto S (2017) Liver transplantation for biliary atresia: a systematic review. Pediatr Surg Int 33:1289–1295. 10.1007/s00383-017-4173-5 28983725 10.1007/s00383-017-4173-5

[12] Sasaki H, Tanaka H, Wada M, Kazama T, Nishi K, Nakamura M, Kudo H, Kawagishi N, Nio M (2014) Liver transplantation following the Kasai procedure in treatment of biliary atresia: a single institution analysis. Pediatr Surg Int 30:871–875. 10.1007/s00383-014-3552-4 25064225 10.1007/s00383-014-3552-4

[13] Sanada Y, Mizuta K, Urahashi T, Ihara Y, Wakiya T, Okada N, Yamada N, Egami S, Ushijima K, Otomo S, Sakamoto K, Yasuda Y, Kawarasaki H (2011) Indication of liver transplantation for jaundice-free biliary atresia with portal hypertension. Ann Transplant 16:7–11. 10.12659/aot.882212 22210415 10.12659/aot.882212

[14] Storey A, McArdle F, Friedmann PS, Jackson MJ, Rhodes LE (2005) Eicosapentaenoic acid and docosahexaenoic acid reduce UVB- and TNF-alpha-induced IL-8 secretion in keratinocytes and UVB-induced IL-8 in fibroblasts. J Invest Dermatol 124:248–255. 10.1111/j.0022-202X.2004.23543.x 15654981 10.1111/j.0022-202X.2004.23543.x

[15] Shirabe K, Bekki Y, Gantumur D, Araki K, Ishii N, Kuno A, Narimatsu H, Mizokami M (2018) Mac-2 binding protein glycan isomer (M2BPGi) is a new serum biomarker for assessing liver fibrosis: more than a biomarker of liver fibrosis. J Gastroenterol. 10.1007/s00535-017-1425-z 29318378 10.1007/s00535-017-1425-z

[16] Wai CT, Greenson JK, Fontana RJ, Kalbfleisch JD, Marrero JA, Conjeevaram HS, Lok AS (2003) A simple noninvasive index can predict both significant fibrosis and cirrhosis in patients with chronic hepatitis C. Hepatology (Baltimore, MD) 38:518–526. 10.1053/jhep.2003.50346 12883497 10.1053/jhep.2003.50346

[17] Yamada N, Sanada Y, Tashiro M, Hirata Y, Okada N, Ihara Y, Urahashi T, Mizuta K (2017) Serum Mac-2 binding protein glycosylation isomer predicts grade F4 liver fibrosis in patients with biliary atresia. J Gastroenterol 52:245–252. 10.1007/s00535-016-1235-8 27349650 10.1007/s00535-016-1235-8

[18] Mo YH, Chen HL, Hsu WM, Chang CH, Peng SS (2021) A noninvasive index to predict liver cirrhosis in biliary atresia. Pediatr Radiol 51:257–264. 10.1007/s00247-020-04823-w 32964265 10.1007/s00247-020-04823-w

[19] Afdhal N, McHutchison J, Brown R, Jacobson I, Manns M, Poordad F, Weksler B, Esteban R (2008) Thrombocytopenia associated with chronic liver disease. J Hepatol 48:1000–1007. 10.1016/j.jhep.2008.03.009 18433919 10.1016/j.jhep.2008.03.009

[20] Hukkinen M, Lohi J, Heikkilä P, Kivisaari R, Jahnukainen T, Jalanko H, Pakarinen MP (2019) Noninvasive evaluation of liver fibrosis and portal hypertension after successful portoenterostomy for biliary atresia. Hepatology communications 3:382–391. 10.1002/hep4.1306 30859150 10.1002/hep4.1306 PMC6396371

[21] Sumida W, Tainaka T, Shirota C, Yokota K, Makita S, Okamoto M, Takimoto A, Yasui A, Takada S, Nakagawa Y, Kato D, Yokoyama S, Ishizu Y, Amano H, Guo Y, Hinoki A, Uchida H (2022) Biochemical markers to predict the development of gastrointestinal bleeding and esophageal varices after portoenterostomy in biliary atresia. Pediatr Surg Int 38:1799–1805. 10.1007/s00383-022-05243-7 36114864 10.1007/s00383-022-05243-7

